# Cardiac revascularization: state of the art and perspectives

**DOI:** 10.1530/VB-19-0011

**Published:** 2019-05-13

**Authors:** Ambra Cappelletto, Serena Zacchigna

**Affiliations:** 1 Cardiovascular Biology Laboratory, International Centre for Genetic Engineering and Biotechnology (ICGEB), Trieste, Italy; 2 Department of Medical, Surgical and Health Sciences, University of Trieste, Trieste, Italy

**Keywords:** heart, revascularization, angiogenesis, arteriogenesis, gene therapy, cell therapy

## Abstract

Cardiac ischemia is the leading cause of morbidity and mortality in a worldwide epidemic. The progressive understanding of the mechanisms driving new blood vessel formation has led to numerous attempts to revascularize the ischemic heart in animal models and in humans. Here, we provide an overview of the current state of the art and discuss the major obstacles that have so far limited the clinical success of cardiac revascularization.

## Mechanisms of vessel formation

Over recent decades, the epidemic of cardiovascular disorders, and in particular cardiac ischemia, has raised an enormous interest on the mechanisms sustaining blood vessel formation, with the final goal of developing novel and effective strategies to revascularize the ischemic heart.

This has led to the identification of three main processes of vessel formation. In the embryo, mesoderm-derived angioblasts differentiate into endothelial cells to form the primitive vascular plexus in a process named vasculogenesis (1). Angiogenesis is the subsequent sprouting of new vascular tubes from pre-existing vessels in response to soluble factors (*in primis* the vascular endothelial growth factor-A, VEGF-A, herein referred as VEGF), the expression of which is induced by tissue hypoxia (2). A subpopulation of endothelial cells (tip cells) guides the sprout toward the source of pro-angiogenic factors, while other endothelial cells (stalk cells) proliferate further to form the tubular structure of the growing vessel. Finally, in adult organisms, physical forces (e.g. shear stress) can remodel and expand pre-existing small vessels to generate larger arteries, in a process known as arteriogenesis (2).

The possible occurrence of a vasculogenesis-like process in adult organisms, driven by circulating endothelial progenitor cells (EPCs) has been postulated over the last 20 years, and is also discussed below. The actual existence of these cells, however, and their functional relevance in inducing effective revascularization of ischemic tissues has been harshly questioned (3).

The progressive understanding of the mechanisms governing blood vessel formation has resulted in attempts to induce the process in the ischemic heart through the delivery of recombinant proteins, genes and cells. In this review, we provide an overview of previous efforts to induce cardiac revascularization and discuss the major obstacles that have so far limited their clinical success.

## Current state of the art

Therapeutic angiogenesis through the delivery of recombinant growth factors, including VEGF, basic fibroblast growth factor (bFGF) and hepatocyte growth factor (HGF), has been successfully achieved in several animal models (4). Yet, the clinical transition of this approach has been largely ineffective, more than likely because of the short half-life of the recombinant proteins that has not allowed for the maturation and stabilization of the newly formed vessels.

Gene-based delivery has been initially proposed as a possible solution to this limitation, considering its potential to drive the persistent production of the therapeutic factor. The first applications of gene therapy for the induction of cardiac therapeutic angiogenesis were centered on the use of VEGF, widely recognized as the most potent angiogenic factor. Again, despite promising pre-clinical success, the clinical experience has been disappointing (5). More recently, alternative genes have been considered. An additional member of the VEGF family, VEGF-D, has gained attention due to its capacity to activate both VEGF receptors 2 and 3, thus simultaneously stimulating angiogenesis and lymphangiogenesis. A phase II clinical trial based on the administration of an adenoviral vector expressing a truncated form of VEGF-D (VEGF-D^∆N∆C^) in patients with *angina pectoris* is currently ongoing (6). In addition to growth factors, recent approaches have explored the angiogenic potential of intracellular proteins, including the G-actin-binding thymosin β4 (Tβ4) and the myocardin-related transcription factor A (MRTF-A) (7, 8). These strategies have shown their efficacy in large animal models of cardiac ischemia, but have never been tested in humans as yet.

Finally, cell therapy has been considered to promote neovascularization and tissue repair after cardiac ischemia. Cells can be either engineered and used as vehicles to deliver growth factors or directly exploited for their pro-angiogenic properties (9, 10). As mentioned earlier, different types of progenitor cells were initially identified for their presumed capacity to sense an ischemic stimulus, home to the site of ongoing neo-angiogenesis and be incorporated into the nascent vessels, directly contributing to the formation of a new vascular network (10). This concept has led to numerous clinical trials aimed at inducing cardiac revascularization through the delivery of EPCs, mesenchymal stromal cells (MSCs), different subpopulation of unselected bone marrow-derived cells (BMCs) and cardiac progenitor cells (CPCs) (11). Overall, these approaches have led to a very modest improvement in cardiac function, which cannot be considered clinically relevant. Furthermore, the capacity of these cells to directly contribute to the neo-vessels through their differentiation into endothelial cells has been challenged (12). Thus, their beneficial effect, if it exists, appears to be largely due to the secretion of growth factors acting in a paracrine manner on resident vascular cells (13). Similarly, the mobilization of stem cells by the delivery of granulocyte-colony stimulating factor (G-CSF) or stromal cell-derived growth factor (SDF-1) did not result in any clinical improvement (14, 15).

## Challenges and future perspectives

Overall, these data indicate that a variety of innovative medical products, including proteins, genes and cells, have been successfully proposed and tested for their capacity to induce cardiac revascularization after ischemia in both small and large animal models. None of these approaches, however, has led to a significant clinical benefit. What could be the reason for these failures?

### Angiogenic factors require appropriate delivery to be effective in inducing functional angiogenesis

As mentioned above, the failure of clinical trials to deliver recombinant proteins by a single injection is most likely attributable to their short half-life and rapid clearance from the tissue. Gene therapy was expected to overcome this limitation, allowing for the continuous production of pro-angiogenic factors (16). Both the route of administration (intramuscular, intracoronary, intrapericardial, intravenous retrograde injection, etc.) and the type of vector are expected to influence the efficiency of gene delivery and past studies are often difficult to compare because they relied on various systems. Initial clinical trials were based on either plasmid DNA or adenoviral vectors, which are fraught by low efficiency and inflammation-driven elimination of transduced cells, respectively. This limited presence of the angiogenic proteins in level and time did not result in effective neovascularization in humans. In parallel, an additional class of viral vectors, derived from the adeno-associated virus (AAV) has emerged and is gaining attention for its capacity to drive persistent transgene expression in the absence of inflammation (17). Experiments performed in animal models have, however, shown that vessels induced by AAV-VEGF are immature and leaky, similar to tumor vessels, and that VEGF expression needs to be finely tuned in time to ensure the generation of a functional vascular network (18, 19). Alternatively, the AAV-mediated combination of VEGF with additional growth factors promoting vessel maturation (e.g. angiopoietin-1 or PDGF-B) has been successfully implemented in experimental animal models, but has never reached clinical application (20, 21). In conclusion, AAV vectors, which also allow the use of inducible or cell-specific promoters, stand as the most promising tools to achieve controlled expression of pro-angiogenic molecules and thus functional revascularization of the ischemic heart.

### Existing animal models do not represent the conditions of patients affected by cardiac ischemia

Small and large animal models of human diseases, including heart ischemia, are extremely useful for both understanding the mechanisms of the disease and testing novel therapies. These animals are often young, do not present additional co-morbidities and share a similar, if not identical, genetic background (22, 23). In contrast, human patients are very heterogeneous in these aspects as well as in the severity of their disease. In addition, end-stage patients, with exhausted angiogenic capacity, are often selected for phase I clinical trial. This might explain, at least in part, why successful experimental strategies have failed when translated to humans and warrant the development of novel models. Experimental research on aged animals is extremely costly and is impossible to perform in most laboratories, requiring animal housing for prolonged periods of time. The execution of pre-clinical trials in aged companion animals, which share most age-related diseases with humans, including cardiac ischemia, could represent a powerful option to screen for the most effective therapies to be brought forward to the clinical phase (while at the same time offering novel therapeutic opportunities to diseased animals) (24). The use of these animals would also allow selecting the most appropriate endpoints (ideally based on current imaging methods such as positron emission tomography and magnetic resonance) to evaluate effective neo-vascularization in future clinical trials.

### Revascularization of the ischemic heart requires a hierarchy of different vessel types

Most of the studies aimed at inducing heart revascularization have focused on the detection of small capillaries, which are assumed to be essential to deliver oxygenated blood to cardiac cells. To be functional, however, capillaries need to be part of a more complex network, also comprising large and small arteries and veins, properly organized in a hierarchical structure. An elegant study based on the genetic tracing of Apelin+ sprouting endothelial cells has recently revealed that the dominant mechanism for the formation of collaterals after cardiac ischemia is arteriogenesis and not angiogenesis (25). This observation opens novel research avenues, as it indicates that cardiac endothelial cells are not able to proliferate and form new capillaries in response to ischemia, but rather the small arterioles that appear at the border of a myocardial infarction derive from pre-existing coronary vessels. The cellular and molecular mechanisms controlling arteriogenesis have been studied quite extensively in models of peripheral ischemia (26) and limited information is available for the heart.

In addition to creating arterioles and capillaries that are able to provide oxygenated blood to cardiac tissue, effective revascularization also implies the formation of vascular structures, such as veins and lymphatics, capable of draining the fluid out of the myocardium. While some studies have started highlighting the relevance of lymphangiogenesis for the therapy of heart ischemia (27), very little is known about the possible induction of venous vessels and their requirement to achieve cardiac revascularization.

### Organotypic features of endothelial cells, an obstacle toward revascularization and regeneration?

The heart is known to be a poorly regenerative organ and this has been largely attributed to the incapacity of adult cardiomyocytes to proliferate in response to damage (28, 29). Over the last decades, it has emerged that, unlike adult mammals, some lower vertebrates and neonatal mice are able to regenerate their myocardium after injury (30, 31). More recently, neovascularization has been recognized to precede, and possibly drive, cardiac regeneration in these models (32).

Emerging evidence indicates that endothelial cells not only provide structural support to blood vessels, but actively control tissue homeostasis during development, disease and regeneration (33). These cells are highly heterogeneous in terms of both transcriptome and functional properties, depending on the tissue in which they are embedded, as shown by their unique transcriptome (34). Cardiac endothelial cells, in particular, are characterized by a unique genetic signature conferring them the ability to uptake and transfer fatty acids to adjacent cardiomyocytes (35, 36). The extent to which these cardiac-specific features also account for the poor angiogenic and regenerative potential of the adult heart is an interesting hypothesis that deserves further investigation.

## Declaration of interest

The authors declare that there is no conflict of interest that could be perceived as prejudicing the impartiality of this review.

## Funding

This work was supported by grant AIRC IG 2016 19032 to S Z.
